# Homogenize Strain Distribution via Molecular Network Engineering for Mechanically Reliable Flexible Perovskite Solar Cells

**DOI:** 10.1007/s40820-026-02079-x

**Published:** 2026-01-26

**Authors:** Fuhao Han, Zuhong Zhang, Hongzhuo Wu, Hongxing Yuan, Linfeng Lu, Zhenhuang Su, Xingyu Gao, Qi Cao, Zhihao Li

**Affiliations:** 1 Key Lab for Special Functional Materials of Ministry of Education, National & Local Joint Engineering Research Center for High-Efficiency Display and Lighting Technology, School of Nanoscience and Materials Engineering, and Collaborative Innovation Center of Nano Functional Materials and Applications, Henan University, Kaifeng, 475004 People’s Republic of China; 2 Henan Key Laboratory of Advanced Conductor Materials, Institute of Materials, Henan Academy of Sciences, Zhengzhou, 450001 People’s Republic of China; 3 Shanghai Synchrotron Radiation Facility (SSRF), Shanghai Advanced Research Institute, Chinese Academy of Sciences, 239 Zhangheng Road, Shanghai, 201204 People’s Republic of China; 4 Huairou Lab, Minist Renewable Energy, Beijing, 101400 People’s Republic of China

**Keywords:** Flexible perovskite solar cells, Homogenize strain distribution, Crosslinking, Mechanical toughness, Crystallization

## Abstract

Flexible perovskite solar cells (FPSCs) suffer from strain localization-induced mechanical degradation, primarily due to heterogeneous strain distribution at grain boundaries. Herein, we propose a molecular engineering approach involving a crosslinked Methacrylic anhydride (MA) to construct a 3D crosslinking network within perovskite films. This molecular-scale network effectively redistributes localized strain into a more homogeneous pattern, as indicated by reduced strain variance and a lower Young’s modulus. Simultaneously, the MA network modulates crystallization kinetics, leading to enlarged grain sizes, enhanced (001) orientation, and decreased defect density. Together, these effects minimize strain concentration and promote elastic strain release, thereby suppressing microcrack formation at grain boundaries. As a result, the optimized rigid perovskite solar cells exhibit superior conversion efficiency of 26.42%, while the FPSCs reach 25.03% with excellent mechanical stability.

**Supplementary Information:**

The online version contains supplementary material available at 10.1007/s40820-026-02079-x.

## Highlights

- Dual-function molecular ligand (MA) can coordinate with Pb^2+^ to passivate defect at grain boundaries and undergoes in-situ polymerization to form a stress-buffering network.
- Attributing to the simultaneous defect suppression and strain homogenization, the MA-modified perovskite solar cells demonstrate high photovoltaic performance with power conversion efficiency up to 26.42% (rigid) and 25.03% (flexible).
- The MA-modified devices demonstrate excellent stability under various environmental stress conditions, including thermal aging, light irradiation, and bending.

**Supplementary Information:**

The online version contains supplementary material available at 10.1007/s40820-026-02079-x.

## Graphical abstract

**Figure:**
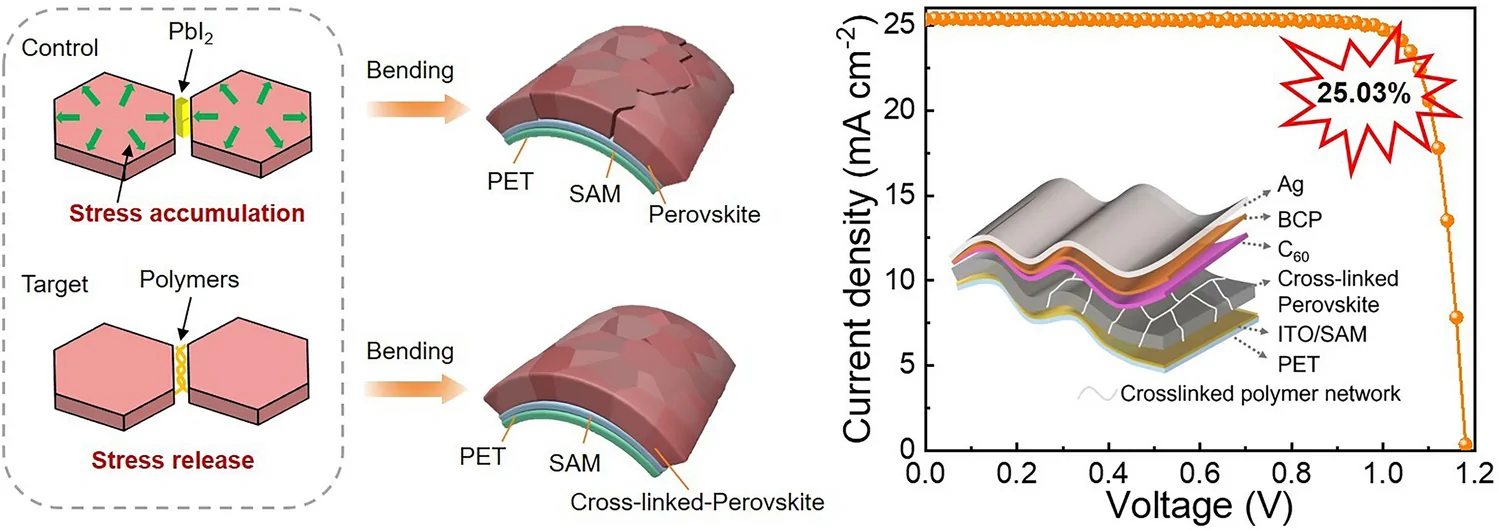

## Introduction

Flexible perovskite solar cells (FPSCs) have attracted significant attention for use in portable electronic, intelligent buildings and unmanned systems, due to their light weight, compatibility with irregular surfaces, and low production cost [1, 2], With ongoing advancements in solvent engineering [3], interface engineering [4–6], component engineering [7], and additive engineering [8–10], the current certified power conversion efficiency (PCE) of FPSCs has transcended from the initial 2.6% to the current 25.44% [11–16], rivaling their rigid counterparts, highlights the immense potential of FPSCs for practical applications. However, for successful commercialization, mechanical reliability is as crucial as high efficiency. The commercial standard for mechanical reliability adopts the commonly used existing ISO protocol, which evaluates flexibility using indicators such as bending cycle count, bending radius, and PCE retained after bending [17, 18]. Although laboratory-scale devices have demonstrated encouraging results, the mechanical stability of FPSCs under combined stressors (e.g., light, moisture, and mechanical deformation) remains a critical challenge for commercial applications.

The inherent brittleness of perovskite crystalline films critically impacts the mechanical durability of FPSCs [19]. In particularly, polycrystalline perovskite films contain numerous grain boundaries (GBs), which serve as defect-rich and degradation-prone regions [20]. On one hand, poor perovskite crystallization on flexible substrates often leads to an even higher density of GBs, increasing the number of defect recombination centers and accelerating photoelectric degradation during bending [21–24]. On the other hand, GBs act as strain concentration points, making the films more susceptible to cracking under mechanical stress, leading to significant decrease in efficiency [25].

In general, two types of strategies have been commonly used to enhance the mechanical stability of F-PSCs [26]: one approach involves forming larger grains sizes with high crystallinity to reduce defect density by introducing functional additives to adjust nucleation and growth process of perovskite [27–29]. The other strategy involves releasing the stress at the GBs by incorporating elastomers or crosslinkable polymers into the perovskite films [30–35]. However, for defect passivation approaches, although it effectively passivating grain boundary defects, it may not address the strain concentration under bending, limiting holistic mechanical robustness. Meanwhile, for strain-alleviation approaches, it may primarily focus on strain distribution might not simultaneously and optimally tackle the defect nucleation at the atomic level, particularly impurities like PbI_2_ at grain boundaries which also induce localized strain [36, 37]. These studies underscore the importance of improving film quality, they predominantly address either defect passivation or strain alleviation in a relatively isolated manner. Therefore, a coupled strategy involve designedly suppresses defect nucleation while simultaneously establishing a robust network to homogenize mechanical stress to achieve mechanical reliability.

In this study, we report a method that can be facilely applied to achieve collective roles of the two strategies mentioned above. Specifically, a liquid cross-linkable Methacrylic anhydride (MA) into the perovskite precursor to inhibit the PbI_2_ impurities nucleated at the contacted GBs and reduce strain accumulation. The carbonyl groups in MA coordinate with Pb^2+^ and promote larger grain growth and enhanced crystallinity, resulting in reduced defect density and improved charge transport. Moreover, in-situ crosslinking forms a robust network that ensures more uniform strain distribution and minimizes stress concentration within the film. As a result, the optimized rigid and flexible PSCs achieved PCEs of 26.42% and 25.03%, respectively. Notably, the flexible devices retained 90% of their initial PCE after 3000 bending cycles, demonstrating excellent mechanical durability.

## Experimental Section

### Materials

I-doped tin oxide (ITO), bathocuproine (BCP, 99.9%) were purchased from Libra Technology Corporation. Lead iodide (PbI_2_, 99.999%), Formamidinium iodide (FAI, 99.99%), methylammonium bromide (MABr, 99.99%) and methylamine chloride (MACl, 99.99%) were purchased from Greatcell Solar (Australia). 2-(3,6-Dimethoxy-9H-carbazol-9-yl) ethyl phosphonic acid (MeO-2PACz, 99.99%) and Lead (II) bromide (PbBr_2_, 99.99%) were purchased form Tokyo Chemical Industry (TCI). Cesium iodide (CsI, 99.999%) was purchased from Xi’an Yuri Solar Co., Ltd., dimethyl sulfoxide (DMSO, 99.7%, SuperDry, with molecular sieves), N, N-Dimethylformamide (DMF, 99.8%, SuperDry, with molecular sieves), Chlorobenzene (CB, 99.8%, SuperDry, with molecular sieves), ethyl alcohol and isopropanol (IPA, 99.5%, SuperDry, with molecular sieves) were purchased from J&K scientific. Methacrylic anhydride (MA, 94%, 0.2% topanol stabilizing agent) was purchased from MACKLIN.

### Device Fabrication

The inverted device architecture was PET (ITO)/SAM/PSK/C_60_/BCP/Ag. The PET (ITO) substrate was treated with UV-ozone for 20 min without cleaning. The SAM solution (0.3 mg mL^−1^ in Absolute ethanol) was spin-coated on PET (ITO) substrate at 3000 rpm for 30 s and then annealed at 100 °C for 10 min. For the preparation of perovskite precursor solution, 1.7 M Cs_0.05_MA_0.05_FA_0.90_PbI_3_ perovskite precursor in DMF: DMSO (4:1 volume ratio, v: v) with 5% MAPbCl_3_ excess. For the doped solution, MA (1 mol L^−1^) was added in previous perovskite solution. The perovskite solution was stirring for 12 h and filtered with a filter head of 0.22 μm. For the perovskite layer, 100 μL prepared perovskite solution was spin-coated on the HTL at 1000 and 5000 rpm for 10 and 30 s with a ramp of 500 and 2000 rpm s^−1^. During the last 10 s of the spinning process, the film was treated by drop-casting chlorobenzene (200 μL). The substrates were annealed on a hot plate at 100 °C for 40 min in nitrogen atmosphere. The solar cells were completed followed by thermally evaporating of C_60_ layer (40 nm), BCP layer (10 nm), and Ag layer (110 nm).

### Characterizations

The mechanism of action was verified between C = O and Pb^2+^ through X-ray photoelectron spectroscopy (XPS) (ESCALAB250Xi, Thermo Fisher Scientific) and middle and far infrared spectrometer (Spectrum 400F, PerkinElmer). X-ray diffractometer (XRD, Rigaku D/MAX-2400 diffractometer). The Kelvin probe force microscopy (KPFM) and Conductive atomic force microscope (C-AFM) were conducted by atomic force microscope (AFM, SPA400, Zeiss). Surface morphology was performed by top view scanning electron microscope (SEM, Zeiss, Supra55, SE2 pattern under 5 kV). Dark current–voltage (*J–V*), space-charge-limited current (SCLC), conductivity and current–voltage (*J–V*, 10 mV s^–1^, 100 mW cm^−2^, standard silicon solar cell calibration) were performed sunlight simulator with a digital source meter. (IVX-50, EnLi Technology, Taiwan). The *N*_t_ can be obtained from equation: *N*_t_ = 2*V*_TFL_εε_0_/*eL*^2^, where *V*_TFL_ is the trap-filled voltage, *e* is the elemental charge and *L* is the distance between the electrodes. Transient photocurrent (TPC) and transient photovoltage (TPV) were performed by transient photocurrent/voltage tester (Shanghai Jinzhu Technology Co., LTD, laser 570 nm). Energy band alignment was obtained from ultraviolet photoelectron spectroscopy (UPS, Specs, PHOIBOS 100, Helium lamp) measurement. Transmittance and absorption were performed by Ultraviolet–visible (UV–vis) absorb spectrum (PE Lambda 950). The incident-photon-to-current efficiency (IPCE) was tested by QE-R of Taiwan enlitechnology. Steady-state photoluminescence (PL) was performed fluorescence spectrophotometer (HORIB-FM-2015). GIWAXS mapping was performed via grazing-incidence wide-angle X-ray scattering (GIWAXS, BL14B1 beamline, Pilatus 2 M detector of the Shanghai Synchrotron Radiation Facility (SSRF) using X-ray with a wavelength of 0.6887 Å under 18 keV). PL mapping measurements were conducted using LSM 980.

### Computational Details

First-principles calculations were performed using density functional theory (DFT) as implemented in the Vienna ab initio Simulation Package (VASP). The projector augmented wave (PAW) method was adopted to describe the electron–ion interactions, and the exchange–correlation energy was treated within the generalized gradient approximation (GGA) using the Perdew-Burke-Ernzerhof (PBE) functional. A plane-wave cutoff energy of 550 eV and a k-point mesh with a reciprocal space density of 0.04 Å^−1^ were employed to ensure convergence. Slab models of the FAPbI_3_ surface were constructed with a vacuum layer of 15 Å to avoid artificial interactions between periodic images. The surface defect formation energies of iodine and lead vacancies were calculated before and after surface passivation to evaluate the passivation effects. The defect formation energy was determined according to E_f_ = E_defect(i or Pb)_−E_perfect_ + E_(i or Pb)_.

For the lead vacancy: Lead's low electronegativity may promote the transfer of electrons from the vacancy region to the oxygen atom of the acyl group in acetic anhydride, resulting in some charge transfer and coordination interaction. For the iodine vacancy: the region near the vacancy may have an electronic deficiency due to the absence of iodine atoms. This electronic deficiency may make the area around the iodine vacancy electron-deficient, thus becoming electrophilic and potentially interacting with electron-rich functional groups, such as the oxygen atom in the acyl group.

At last, we position the target molecule near the defect and perform geometric optimization to ensure that the relative orientation between the molecule and the defect corresponds to the lowest energy configuration. This process facilitates the rational placement of the target molecule in the vicinity of the defect, leading to the formation of a stable adsorption structure.

### Bending Durability Tests

The bending durability of FPSCs was evaluated using a mechanical tester (PR-BDM-100, Puri, China) in constant-radius bending mode (30 cycles per minute) with different bending radii (∞, 10, 8, 6, 4, and 2 mm) and bending times (0–3000 times) under room temperature (25–30 °C). The corresponding J-V curves were periodically measured in a flat state under AM 1.5 G100 mW cm^−2^ illumination for 200 cycles.

### Lattice Strain Calculation

For assessing the strain index σ of perovskite lattices, we utilize the 2θ-sin2φ method associated with Bragg’s Law and generalized Hooke’s Law, as Eq. (1):

1 $$\sigma = \frac{E}{{\left( {1 + \nu } \right)sin^{2} \varphi }} \cdot \left( {\frac{{d_{\varphi } - d_{n} }}{{d_{n} }}} \right)$$

where *φ* and *n* are the scattering vector angles, pertaining to the perovskite film surface normal direction.

For the out-of-plane and in-plane directions, we obtain that out-of-plane *φ* = 90° (⊥) and in-plane *n* = 0° (∥). Then it can be calculated that sin^2^*φ* = 1, as shown in Eq. (2):

2 $$\sigma = \frac{E}{{\left( {1 + \nu } \right)}} \cdot \left( {\frac{{d_{ \bot } - d_{\parallel } }}{{d_{\parallel } }}} \right)$$

Due to *q* = 2π/*d*, we further convert Eqs. (2)–(3) as follows:

3 $$\sigma = \frac{E}{{\left( {1 + \nu } \right)}} \cdot \left( {\frac{{q_{\parallel } - q_{ \bot } }}{{q_{\parallel } }}} \right)$$

We introduce ∆*q* to represent the difference between ${q}_{\parallel }$ and ${q}_{\perp }$ ($\Delta q={q}_{\parallel }-{q}_{\perp }$). Since the change in the denominator ${q}_{\perp }$ is negligible compared to that in the numerator ∆*q*, we can use a constant value ${q}_{0}$ to represent, resulting as Eq. (4):

4 $$\sigma = \frac{E}{{q_{0} \left( {1 + \nu } \right)}} \cdot \Delta q$$

where *E* is Young’s modulus and *v* is Poisson’s ratio of the perovskite film, respectively. *E* and *v* are evaluated as 10 GPa and 0.3, respectively. Also, the scattering vector constant* q*_0_ = 10 nm^−1^ is available.

From the resultant Eq. (4), we can observe that *σ* is proportional to ∆*q* value. The ∆*q* is calculated by subtracting the out-of-plane scattering vector *q*_*z*_ value from that (*q*_*y*_) of in-plane.

## Results and Discussion

### Chemical Interaction and Defect Passivation

**Fig. 1:**
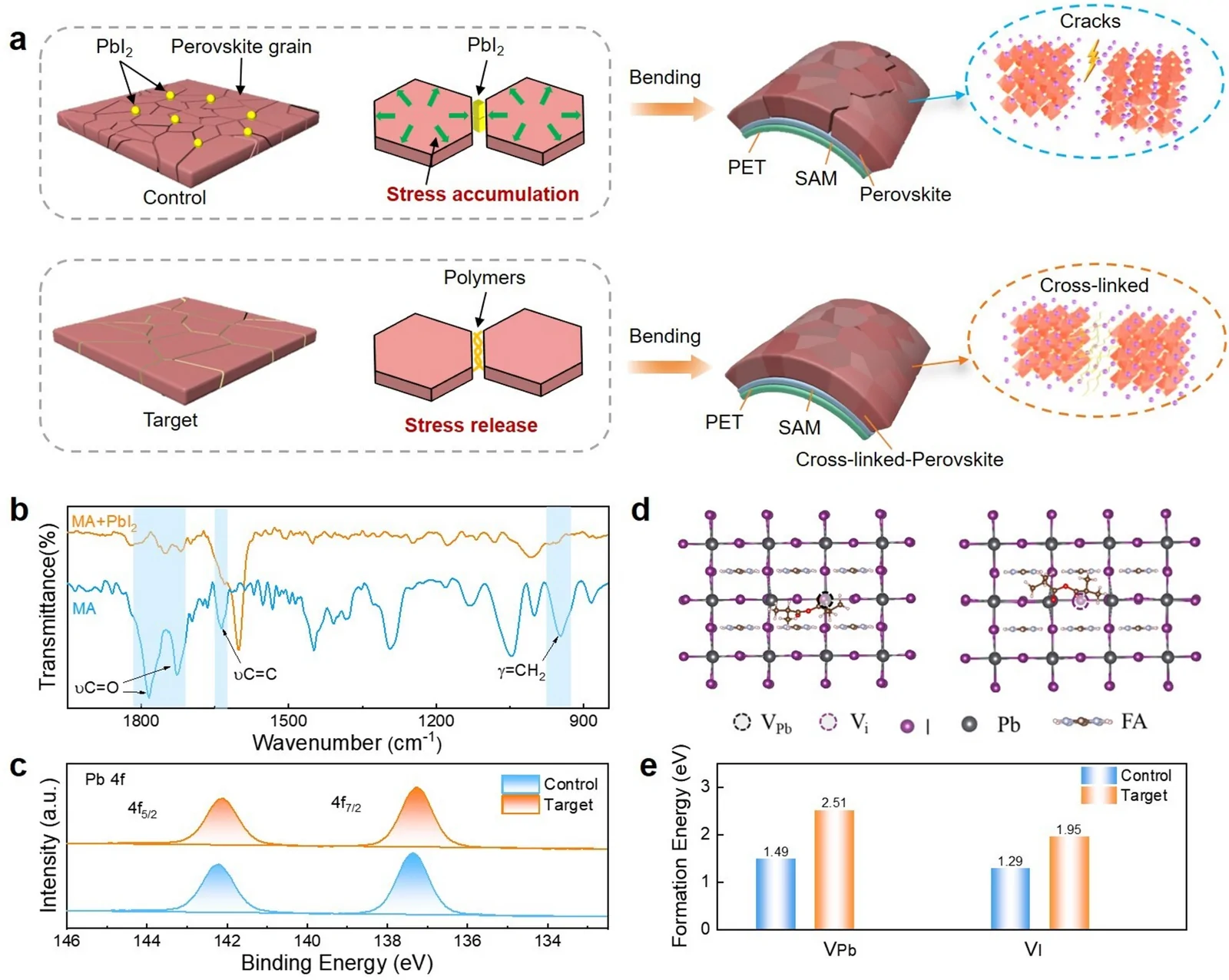
Cross-linking effect of MA in perovskite film. **a** Schematic illustration of MA in perovskite film to release stress and enhance bending resistance. **b** FTIR spectra of pure MA and PbI_2_ film with MA modification after annealing. **c** XPS Pb 4*f* binding spectra of control and MA-modified perovskite films. **d** Top view of the theoretical models of V_Pb_ and V_I_ defects of MA-modified film. **e** Formation energy of V_Pb_ and V_I_ defects before and after passivation by MA

The schematic diagram of the chemical structure of MA cross-linked molecular monomer and its regulation of perovskite crystal growth process is shown in Fig. 1a. The MA molecule is a polymerizable and small molecule monomer with C = C bond at both ends. After annealing, the solution of pure MA changed from transparent to colloidal indicating the formation of cross-linked MA (Fig. S1) [38]. Furthermore, the in-situ cross-linking of MA and the chemical interactions between the cross-linked polymers and perovskites were investigated by Fourier transform infrared spectroscopy (FTIR). As shown in Fig. 1b, the characteristic C = C stretching peak at 1635 cm^−1^ and the bending peak at 949 cm^−1^ almost disappears, indicating that the MA molecule has undergone heat-induced polymerization to form cross-linked polymer networks [39]. In addition, the C = O stretching vibration peaks shift to lower wavenumbers of 1749 and 1718 cm^−1^, which may be induced by the interaction between C = O bonds and Pb^2+^ ions [40, 41]. X-ray photoelectron spectroscopy (XPS) was used to further probe the interactions between MA and perovskite (Fig. 1c). After the modification of MA, the main peaks at 142.20 eV (Pb 4*f*_5/2_) and 137.35 eV (Pb 4*f*_7/2_) were shifted toward low binding energy regions (142.10 and 137.25 eV, respectively), which indicates that the C = O of MA molecule coordinated with Pb^2+^ [42]. The cross-sectional high-resolution TEM image showed the MA cross-linked perovskite film can clearly distinguish the grain boundaries (GBs) between the perovskite grains (Fig. S2). Subsequently, we calculated the formation energies of dominant defects such as iodine vacancy (V_I_) and lead vacancies (V_Pb_) in the perovskite films, using density functional theory (DFT) [43]. Top views of these intrinsic defects and their passivated configurations with MA were presented in Figs. 1d and S3. After being treated by MA, the formation energies of V_Pb_ and V_I_ increased notably from 1.49 and 1.29 eV to 2.51 and 1.95 eV (Fig. 1e), respectively, indicating that MA significantly inhibit the defect formation, thereby facilitating the realization of high-quality perovskite films with enhanced properties.

### Crystallization and Morphology of Perovskite Films

**Fig. 2:**
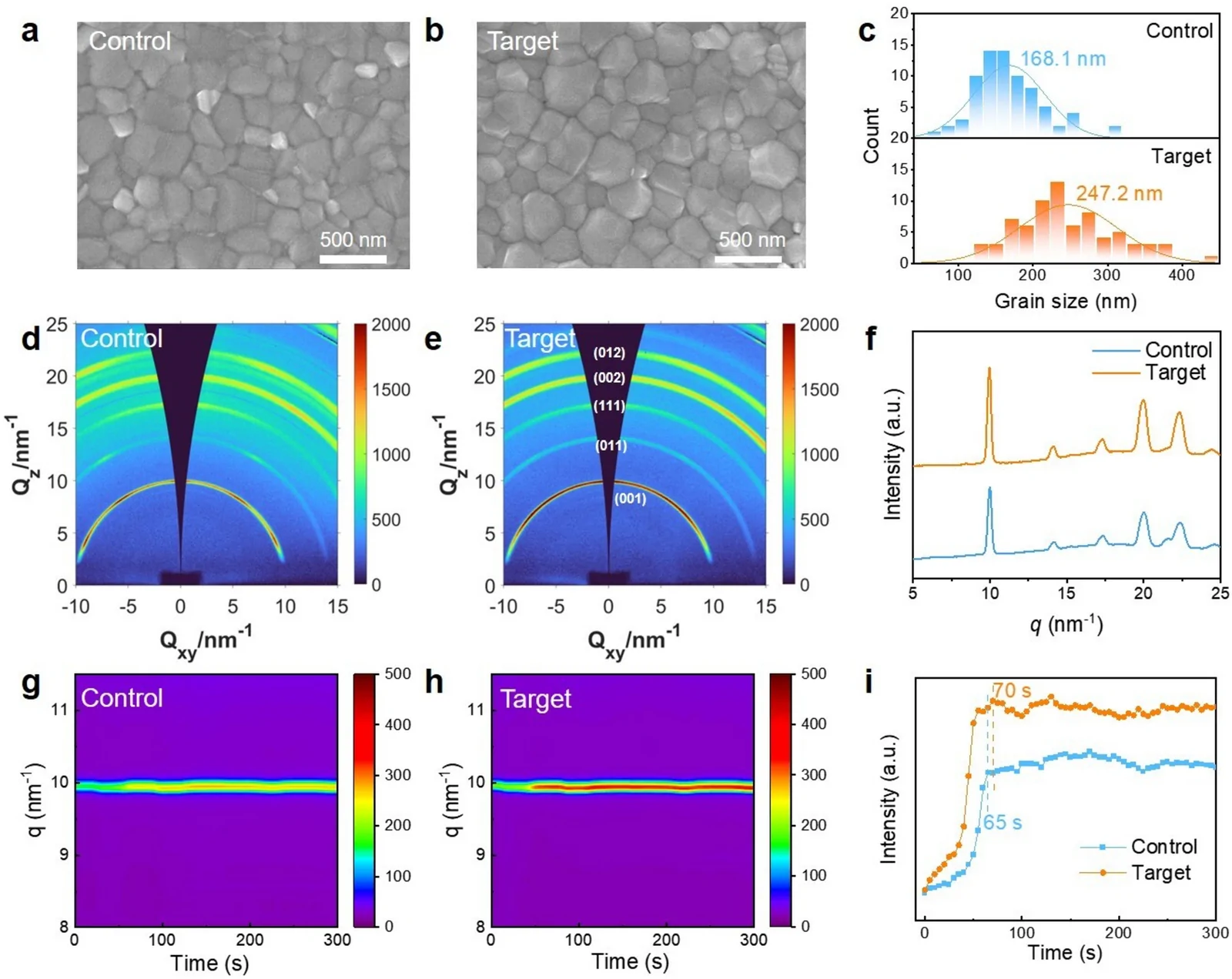
Crystallization kinetics and crystal orientation. The SEM images of **a** control and **b** MA-modified perovskite films. **c** Histogram of grain size distribution corresponding to the top-view SEM. GIWAXS patterns for **d** control and **e** MA-modified perovskite films. **f** GIWAXS *q*-integrated intensity curves for control and MA-modified perovskite films. In situ GIWAXS of the intensity of *q* integrating with times changes in **g** control and **h** MA-modified films. **i** Peak intensity changes with time along with *q* = 10 nm^−1^ from **g** and **h**

To investigate the influence of MA on the crystallization and microstructure of perovskite, scanning electron microscopy (SEM) measurements was conducted. The incorporation of the crosslinked polymer clearly affects film formation, as evidenced by the SEM images. As shown in Fig. 2a, b, the target films exhibit smoother, more uniform surfaces with fewer defects compared to the control, indicating that MA effectively regulates crystal growth. In addition, the average grain size in the target films is larger than in the control film (Fig. 2c). Beyond morphology improvements, grazing-incidence wide-angle X-ray scattering (GIWAXS) data (Fig. 2d–f) show that the target films possess stronger diffraction intensities, suggesting enhanced crystallinity [44]. Due to the strong coordination effect between MA and Pb^2+^, MA may weaken the coordination interaction between PbI_2_ and DMF, resulting in larger colloids (Fig. S4). In situ UV–vis spectra of the perovskite films during annealing indicated that the presence of MA can delay conversion from the intermediate phase to α-FAPbI_3_, which slows the crystallization process and results in larger grain sizes (Fig. S5). To further examine the crystallization process, in-situ GIWAXS was performed, capturing the Q-integrated intensity distribution from 0 to 300 s (Fig. 2g–i). The full spectral data are provided in Fig. S6. Notably, the peak intensity of the (001) plane at *q* = 10 nm^−1^ is significantly larger in the target film than in the control (Fig. 2i), confirming the improved crystallinity induced by MA. And the crystallization time is also increased from 65 to 70 s, indicating that MA polymer can delay the crystallization of perovskite, which further confirms the enhancement of crystallinity.

### Strain Homogenize and Residual Stress Investigation

**Fig. 3:**
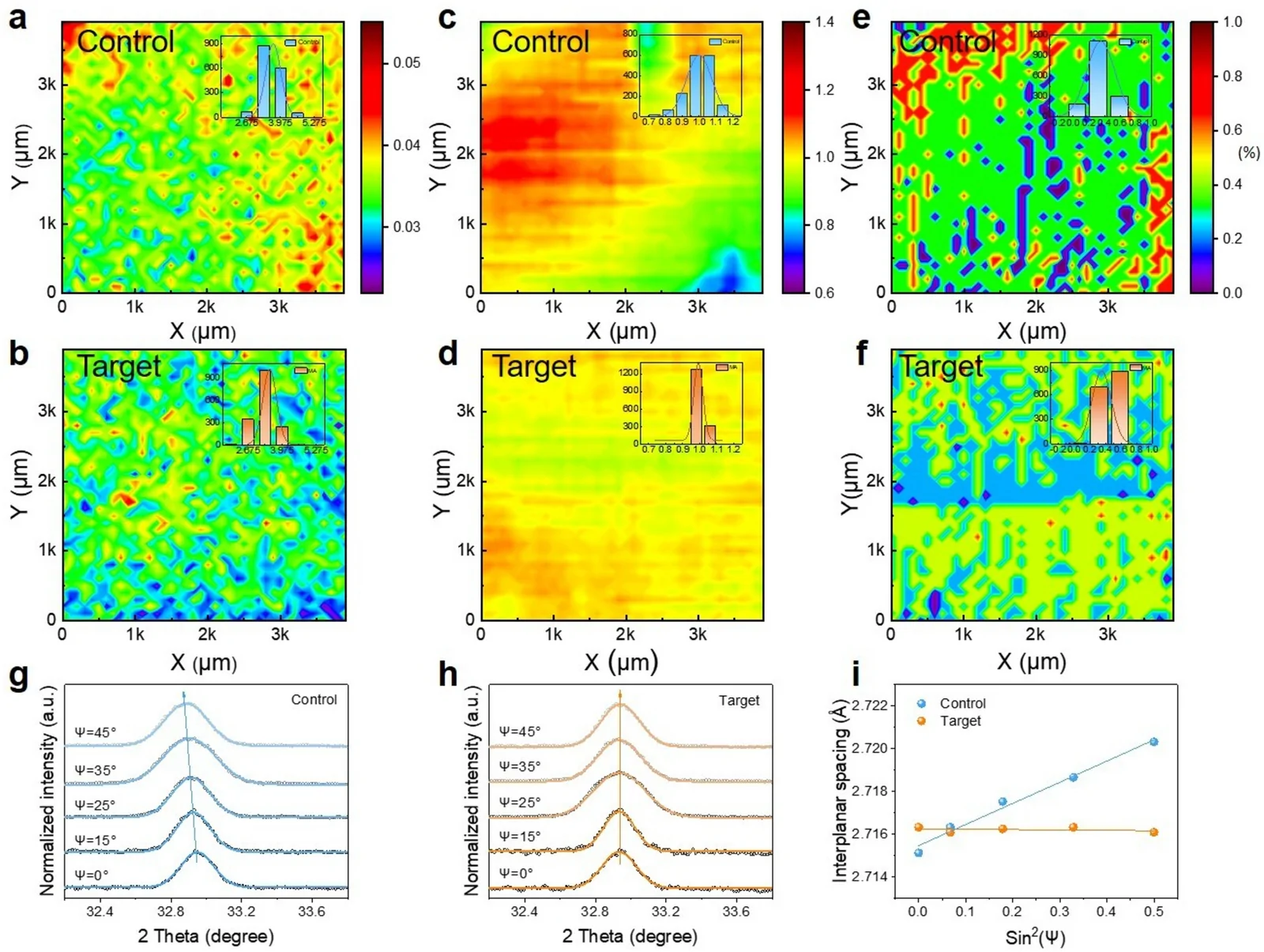
GIWAXS mappings of perovskite films. The distribution image of peak area ratio of PbI_2_ to the perovskite (001) plane in **a** control and **b** target perovskite films. The normalized distribution image of the perovskite (001) plane peak area in **c** control and **d** target films. The normalized distribution image of strain peak area for **e** control and **f** target films. Grazing incidence X-ray Diffraction (GIXRD) patterns at different Ψ angles (from 0° to 45°) for **g** control and **h** target perovskite films. **i** Lattice spacing versus sin^2^(Ψ) plots for control and MA-modified perovskite films

To investigate the surface composition and residual strain distribution in flexible perovskite films, grazing-incidence wide-angle X-ray scattering (GIWAXS) was performed. The peak-area ratios of PbI_2_ to perovskite, overall perovskite peak intensities and strain-related signals were calculated from the Q-integrated data, with the corresponding mapping images presented in Fig. 3. A schematic of the measurement principle is shown in Fig. S7. From the mapping of the PbI_2_-to-perovskite (001) peak-area ratio (Fig. 3a, b), the variance in the target film is 1.38 × 10^–5^, slightly lower than the control film’s 1.40 × 10^–5^ (Fig. S8). This suggests a more uniform distribution of residual PbI_2_ on the target film surface, which could help reduce defect density and suppress non-radiative recombination [45, 46]. The perovskite peak-area mapping (Fig. 3c, d) reveals a substantial improvement in uniformity. The target film shows a variance of 5.56 × 10^–4^, significantly lower than the control film’s 57.58 × 10^–4^, indicating enhanced crystallinity and homogeneity. Similarly, the strain mapping (Fig. 3e, f) shows reduced variance in the target film (1.52 × 10^–2^) compared to the control (3.25 × 10^–2^), confirming that MA minimizes strain concentration and promotes more uniform stress distribution during in-situ crosslinking. The local mechanical properties of perovskite films were quantitatively characterized with the nano-indentation measurement (Fig. S9). It clearly shown that the Young’s modulus of control film is larger and uneven distribution, while the MA-modified film exhibited minor changes. It has been confirmed in detail that the robust cross-linked network formed by MA can form a more uniform strain distribution and minimize stress concentration. Mechanical properties were further evaluated using peak force quantitative nanomechanical (PF-QNM) atomic force microscopy (AFM), as shown in Fig. S10. The average Young’s modulus of the target film is reduced to 8.97 GPa, compared to 11.33 GPa in the control, indicating that the MA polymer enhances film toughness by increasing mechanical flexibility. Grazing-incidence X-ray diffraction (GIXRD) measurements were performed to further elucidate the strain distribution within the perovskite films. As demonstrated in Fig. 3g–i, the diffraction peak of control film gradually leftward shift to lower diffraction angles with increasing Ψ angle (0° to 45°), while the MA-modified film exhibited negligible peak shift, indicating that uniform lattice parameters throughout the film depth and effective strain relaxation.

### Carrier Dynamics Investigation

To further investigate the effect of MA on electrical performance, conductive atomic force microscopy (c-AFM) was conducted (Fig. S11). The target perovskite film exhibits significantly higher average current compared to the control film, which favors excellent carrier transport. Photoluminescence (PL) and time-resolved photoluminescence (TRPL) spectroscopy were employed to study the photoelectric properties and charge carrier dynamics (Fig. S12). The target perovskite film demonstrates significantly higher PL intensity than the control, suggesting suppressed non-radiative recombination [47]. Specifically, the TRPL decay curves showed that the average PL lifetime increases from 243.2 ns in the control to 519.1 ns in the target film (Table S1), further confirming reduced trap density and improved film quality induced by stress release [48]. Trap density was analyzed using the space-charge-limited current (SCLC) method in hole-only and electron-only device configuration (Fig. S13). The target device exhibits lower trap density. This reduction is attributed to effective defect passivation via chemical interactions [49]. Current density–voltage (*J-V*) characteristics measured under dark conditions (Fig. S14) reveal a lower dark current in the target PSCs, suggesting reduced trap-assisted recombination and leakage current [50, 51]. Furthermore, transient photocurrent (TPC) and transient photovoltage (TPV) measurements (Fig. S15) show that the target device has a longer charge carrier lifetime and a shorter photocurrent decay time due to the tensile strain gradient was almost eliminated, indicating more efficient charge extraction and fewer trap states These results confirm that the in-situ crosslinked MA polymer enhances carrier dynamics and reduces recombination losses in perovskite film [52]. Additionally, the target film exhibits a higher surface potential (Fig. S16) [53]. This enhanced surface potential further supports improved charge transfer and reduced carrier recombination in the MA-modified perovskite film.

### Photovoltaic Performance and Stability

**Fig. 4:**
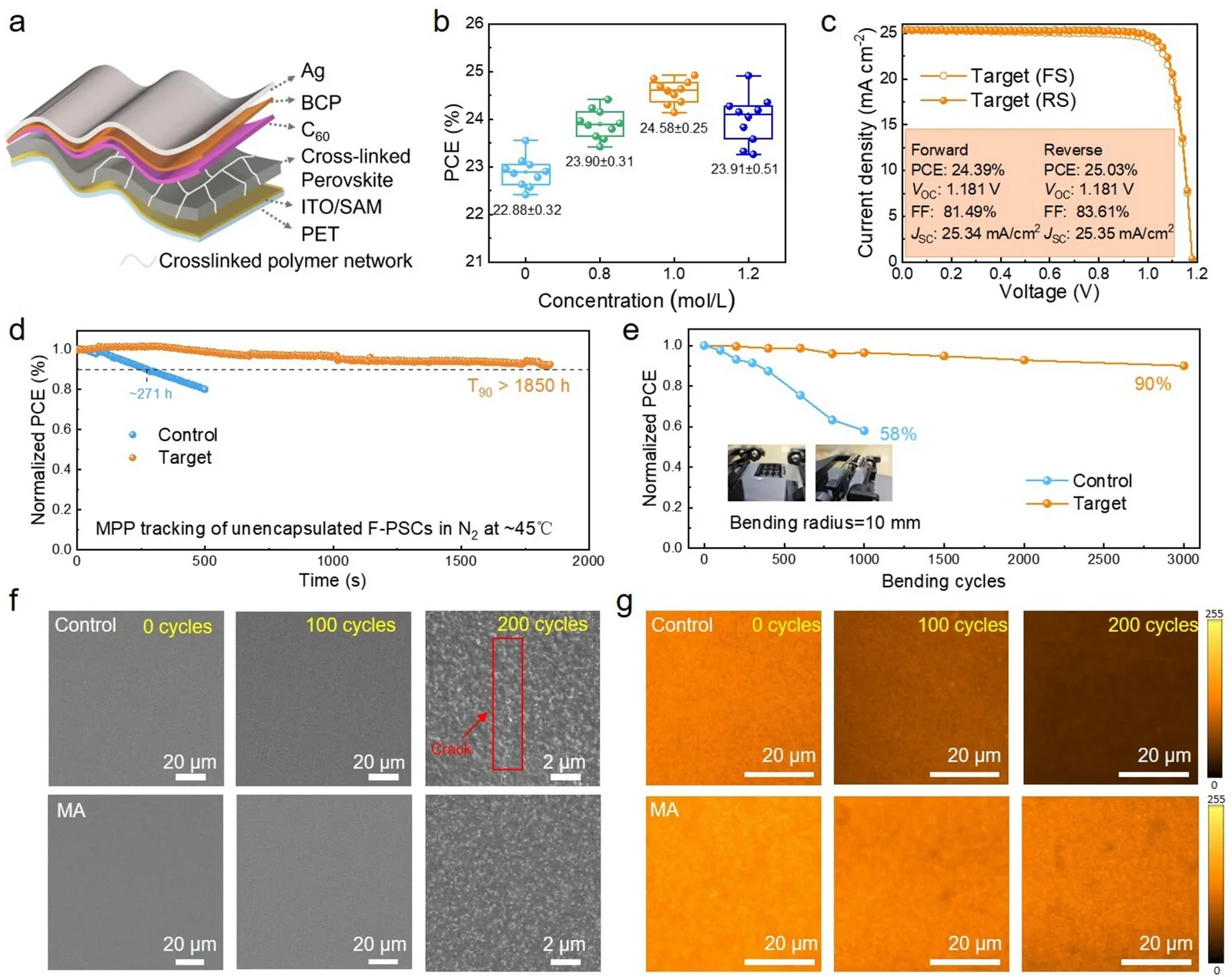
Photovoltaic performance and mechanical stability of flexible PSCs. **a** Schematic diagram of flexible device structure. **b** PCE histogram of FPSCs at different concentration. **c** *J-V* curves of target device. **d** Continuous maximum power point (MPP) tracking of unencapsulated FPSCs in nitrogen atmosphere. **e** Normalized PCE of the FPSCs after 3000 bending cycles at a curvature radius of 10 mm. **f** SEM images of flexible perovskite films in unbending and bending 200 cycles at a radium of 5 mm. **g** PL mapping of perovskite films after different bending cycles at a curvature radius of 5 mm

Based on the above findings, FPSCs were fabricated with the device structure PET/ITO/SAM/perovskite/C_60_/bathocuproine (BCP)/Ag, as illustrated in Fig. 4a. The effect of MA concentration on device performance was systematically studied (Figs. 4b and S17), with 1.0 mol L^−1^ identified as the optimal concentration. The *J-V* curve of the optimized FPSC was shown in Fig. 4c. Compared to the control device (Fig. S18), the MA-modified device achieved a champion PCE of 25.03% with *V*_OC_ of 1.18 V, *J*_SC_ of 25.35 mA cm^−2^ and fill factor of 83.61%. The *J*_*SC*_ derived from the *J-V* measurement aligns well with the integrated current density obtained from the external quantum efficiency (EQE) spectrum (Fig. S19)**.** The operational stability was tested at maximum power point under continuous illumination in N_2_ atmosphere. The MA-modified PSCs remained 90% of its original efficiency after 1850 h due to the defect passivation and strain release effect, while the PCE of control device declined considerably (Fig. 4d). Additionally, the MA additive was applied in rigid device, demonstrating superior performance, and achieving a champion PCE of 26.42% (Fig. S20). Furthermore, device stability was evaluated under nitrogen atmosphere at room temperature. The unencapsulated flexible MA-modified device retained over 89% of its initial PCE after 6000 h of storage, whereas the control device retained only 53% (Fig. S21). Thermal stability tests were conducted on devices under the ISOS-D-2 protocol. As shown in Fig. S22, the MA-based device retained 94.2% of its initial PCE after 500 h in a nitrogen environment at 85 °C, whereas the control device exhibited rapid degradation over time, confirming the excellent stability imparted by the MA additive.

Finally, the mechanical stability of the FPSCs was evaluated through bending tests. As shown in Fig. 4e, the MA-modified device retained 90% of its initial PCE after 3000 bending cycles at a bending radius of 10 mm, while the control device maintained only 58% of its initial efficiency after 1000 cycles under the same conditions. Figure S23 further illustrates the performance under different bending radii. After 1000 cycles at a severe bending radius of 2 mm, the control device showed a 63% loss in PCE, whereas the target device retained 81% of its initial performance, demonstrating significantly enhanced mechanical robustness due to the in-situ crosslinking effect of MA. To further verify these results, SEM images were captured before and after 200 bending cycles at a radius of 5 mm (Figs. 4f and S24). Pronounced cracking was observed in the perovskite layer of the control film after bending, while the MA-modified film remained largely intact, exhibiting minimal visible damage. These results confirm that MA significantly enhances the mechanical integrity of the flexible perovskite film under repeated deformation. To further elucidate the mechanism behind the enhanced stability imparted by MA, steady-state photoluminescence (PL) mapping was conducted to monitor the evolution of film morphology and optoelectronic properties under mechanical stress. PL intensity mapping (Fig. 4g) reveals significantly higher PL intensity in the target film compared to the control, consistent with the previous steady-state PL results. This suggests reduced nonradiative recombination and improved crystallinity in the MA-treated films. Notably, after 200 bending cycles, the target film retains 74% of its initial PL intensity, whereas the control retains only 35%. This substantial difference reflects enhanced carrier transport stability and provides further evidence of the mechanical reinforcement achieved through in-situ crosslinking.

## Conclusion

In summary, a cross-linkable molecule (MA) was incorporated into the perovskite precursor to enhance the mechanical robustness of perovskite films. The C = O groups in MA coordinate with undercoordinated Pb^2+^ ions, effectively passivating lead-related defects, resulting in the formation of high-quality and large-grain perovskite films. Through in-situ crosslinking, MA forms a robust polymer network at grain boundaries, which reduces residual strain and improves strain distribution across the film. As a result, MA-modified flexible and rigid devices achieve champion PCEs of 25.03% and 26.42%, respectively, along with outstanding mechanical durability. This study presents an effective strategy for improving the mechanical toughness of FPSCs and offers valuable insights into mitigating mechanical degradation in flexible perovskite devices.

## Supplementary Information

Below is the link to the electronic supplementary material.

Supplementary file1 (DOCX 6105 kb)
