# Supplementary material for: Homogenize Strain Distribution via Molecular Network Engineering for Mechanically Reliable Flexible Perovskite Solar Cells
Source: Nanomicro Lett. 2026 Jan 26;18:218. doi: 10.1007/s40820-026-02079-x (PMC12832598; doi:10.1007/s40820-026-02079-x)
Supplement: Supplementary file 1 — Supplementary file1 (DOCX 6105 kb) [file 40820_2026_2079_MOESM1_ESM.docx]

Supporting Information for

**Homogenize Strain Distribution via Molecular Network Engineering for Mechanically** **Reliable Flexible Perovskite Solar Cells**

Fuhao Han^1^, Zuhong Zhang^1^, Hongzhuo Wu^1^*, Hongxing Yuan^2^, Linfeng Lu^3^, Zhenhuang Su^3^*, Xingyu Gao^3^, Qi Cao^4^*, Zhihao Li^1^*

^1^ Key Lab for Special Functional Materials of Ministry of Education, National & Local Joint Engineering Research Center for High-efficiency Display and Lighting Technology, School of Nanoscience and Materials Engineering, and Collaborative Innovation Center of Nano Functional Materials and Applications, Henan University, Kaifeng 475004, P. R. China

^2^ Institute of Materials, Henan Key Laboratory of Advanced Conductor Materials, Henan Academy of Sciences, Zhengzhou 450001, P. R. China

^3^ Shanghai Synchrotron Radiation Facility (SSRF), Shanghai Advanced Research Institute, Chinese Academy of Sciences, 239 Zhangheng Road, Shanghai 201204, P. R. China

^4^ Minist Renewable Energy, Huairou Lab, Beijing 101400, P. R. China

*Corresponding authors. E-mail: [wuhongzhuo@henu.edu.cn](mailto:wuhongzhuo@henu.edu.cn) (Hongzhuo Wu); [suzh@sari.ac.cn](mailto:suzh@sari.ac.cn) (Zhenhuang Su); [2022031059@nwpu.edu.cn](mailto:2022031059@nwpu.edu.cn) (Qi Cao); [lizhihao@henu.edu.cn](mailto:lizhihao@henu.edu.cn) (Zhihao Li)

**Supplementary Figures and Tables**

**Fig. S1** The polymerization process of MA molecules and corresponding photos

**Fig. S2** Cross-sectional transmission electron microscopy (TEM) images of MA-modified PSCs

**Fig. S3** Top view of the theoretical models of V_Pb_ and V_I_ defects of control film


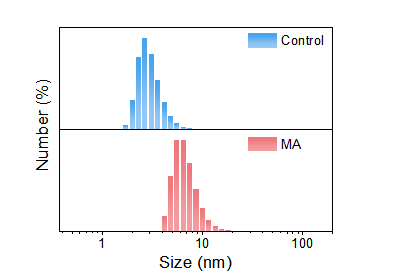


**Fig. S4** Colloidal size distribution of precursor solution without and with MA


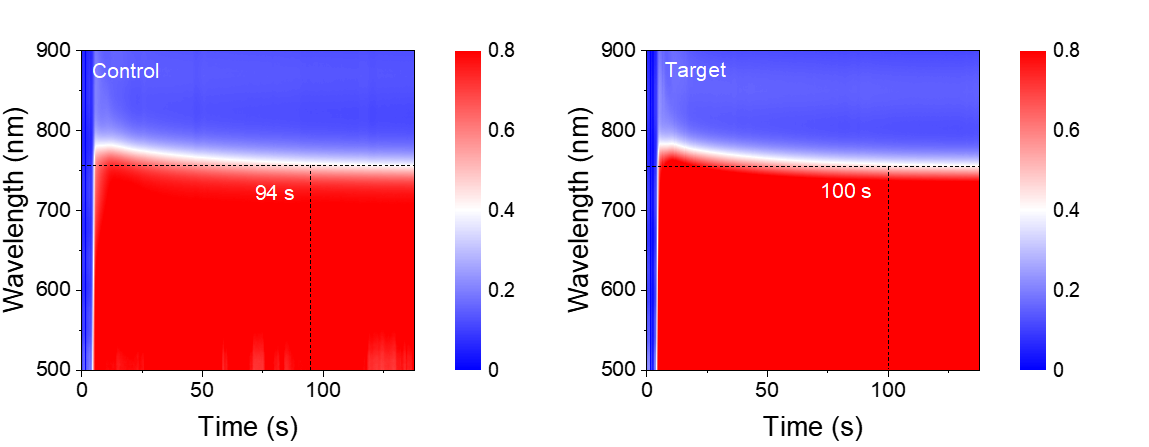


**Fig. S5** In situ absorption spectra of control and target perovskite films


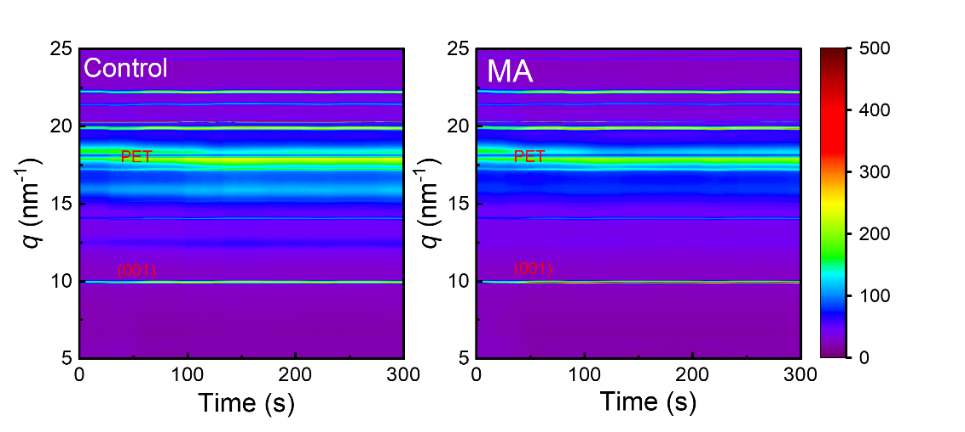


**Fig. S6** In situ GIWAXS images of the intensity of *q* integrating with times changes in the control and MA modified films


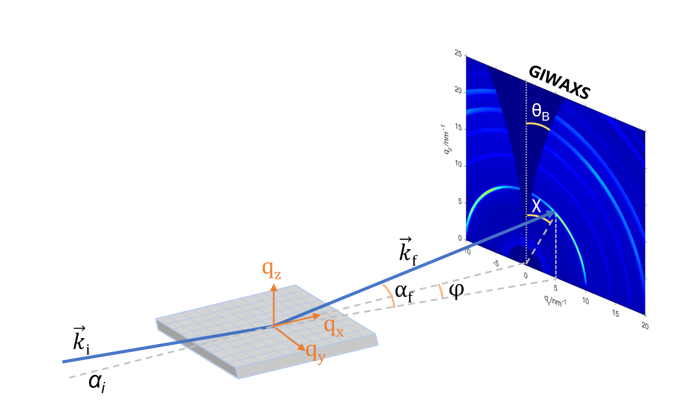


**Fig. S7** Schematic diagram of GIWAXS test


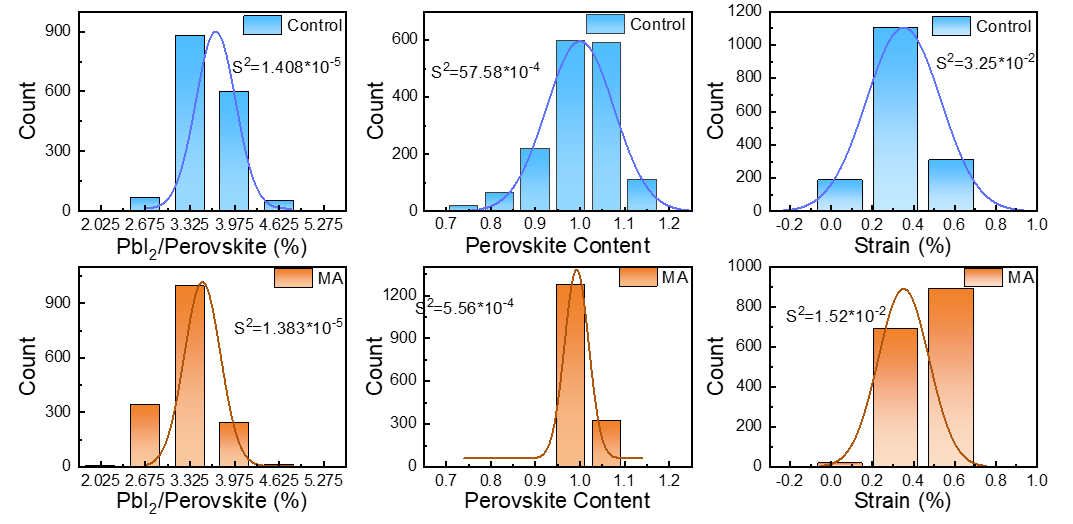


**Fig. S8** The statistical distribution chart of the peak area from GIWAXS mapping of the flexible perovskite films


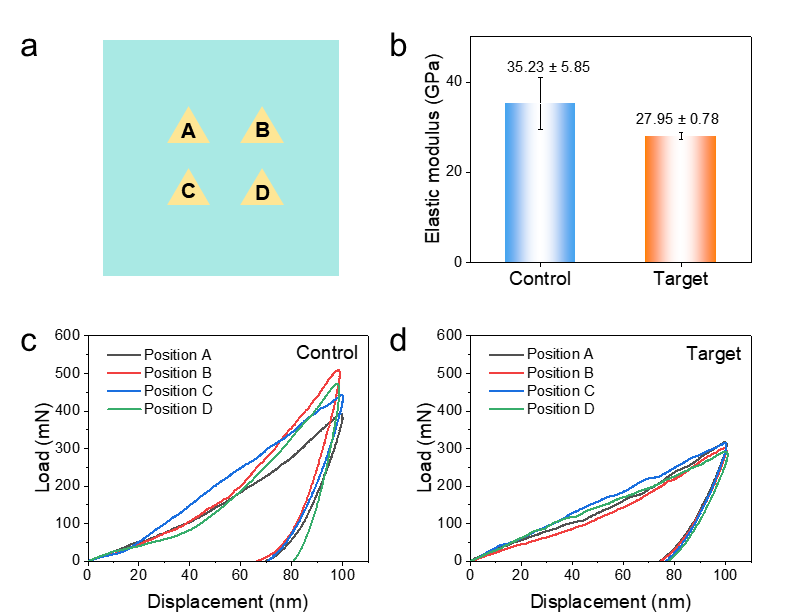


**Fig. S9** **a** Schematic diagram of nanoindentation array. **b** Elastic modulus of control and MA-modified perovskite film. Representative load-displacement curve of indentation test for **c** control and **d** MA-modified perovskite film


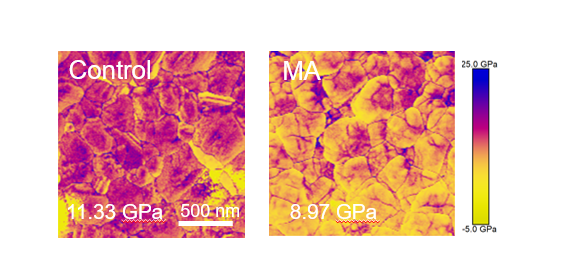


**Fig. S10** The Young's modulus diagram of flexible perovskite film

**Fig. S11** Surface topography and current of AFM

**Fig. S12** **a** Steady-state PL curves and **b** TRPL decay curves of control and MA-modified perovskite film


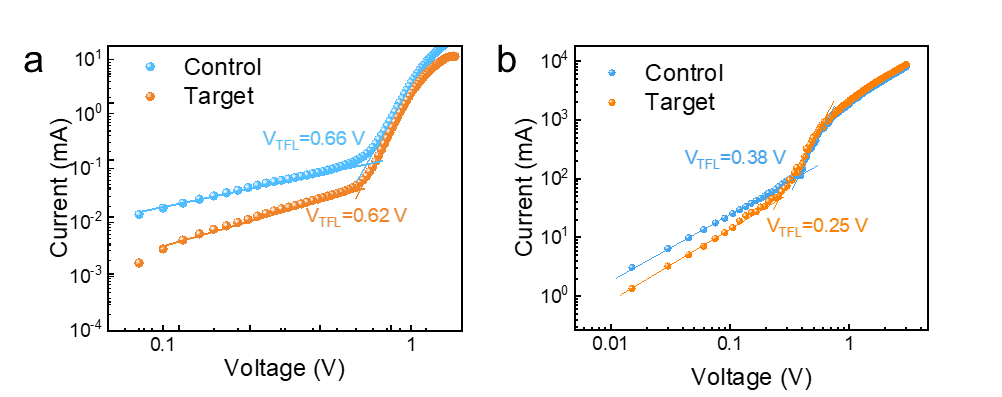


**Fig. S13** Current-voltage curves of **a** hole-only and **b** electron-only devices via the SCLC method


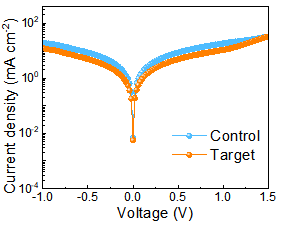


**Fig. S14** The dark J-V curves of PSCs

**Fig. S15** Transient photovoltage and transient photocurrent decay of the control and target devices


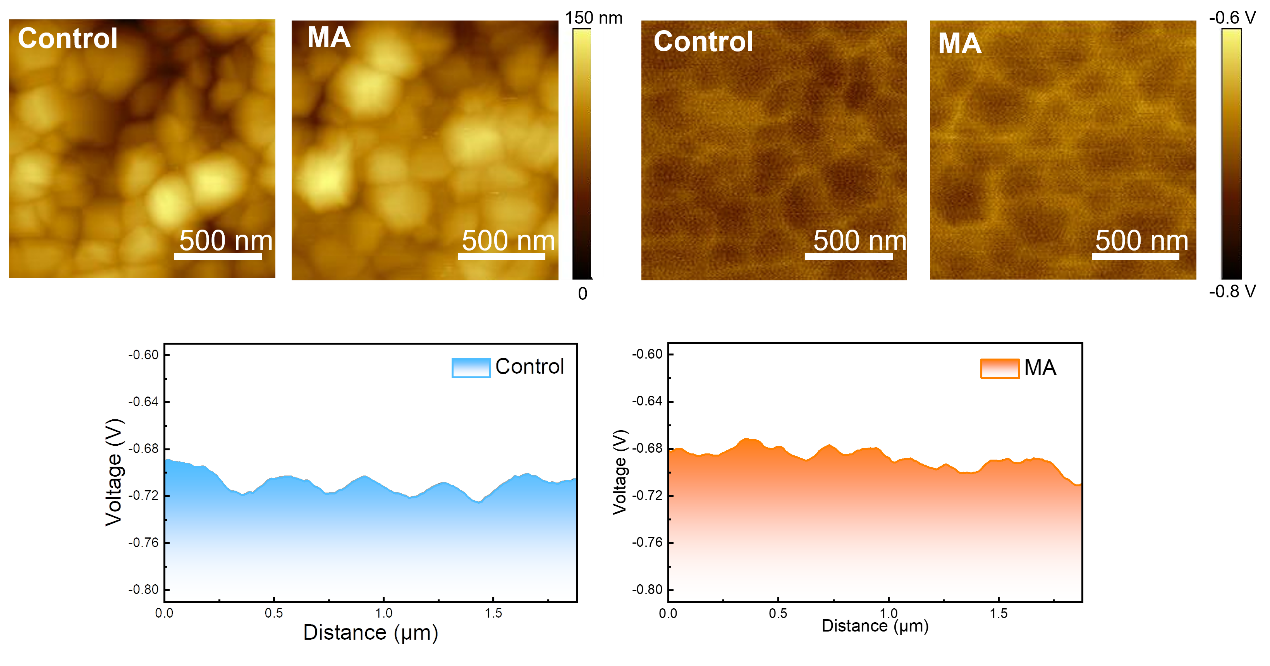


**Fig. S16** The surface potential diagram of perovskite film in AFM

**Fig. S17** Photovoltaic parameter statistics of control and target FPSCs at different concentration: **a** V_OC_; **b** J_SC_; **c** FF; **d** PCE


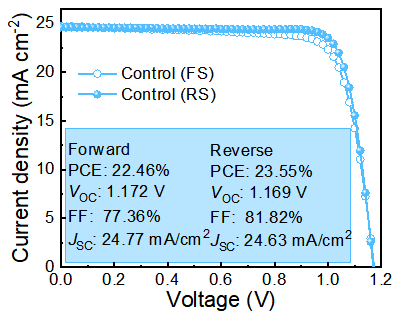


**Fig. S18** The *J-V* curves of control flexible device


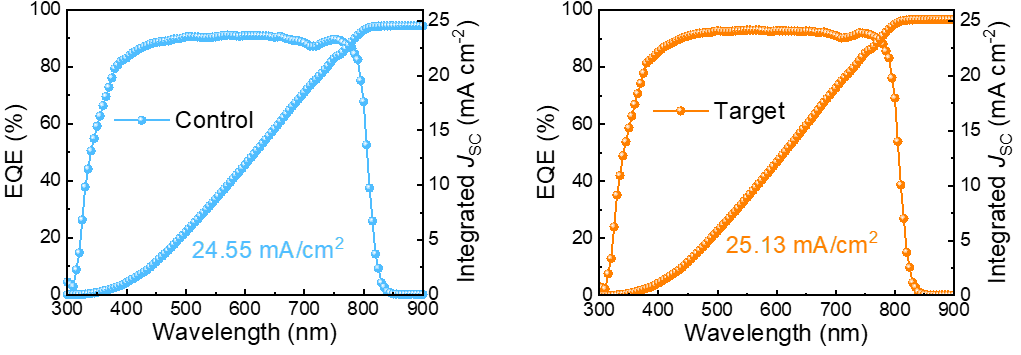


**Fig. S19** The EQE spectra and integrated *J_SC_* of the control and target FPSCs


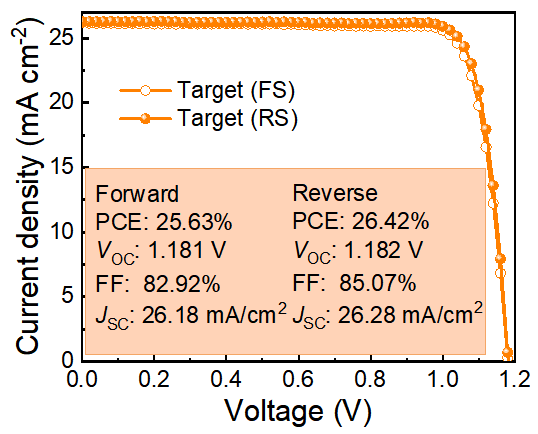


**Fig. S20** *J-V* curves of rigid device

**
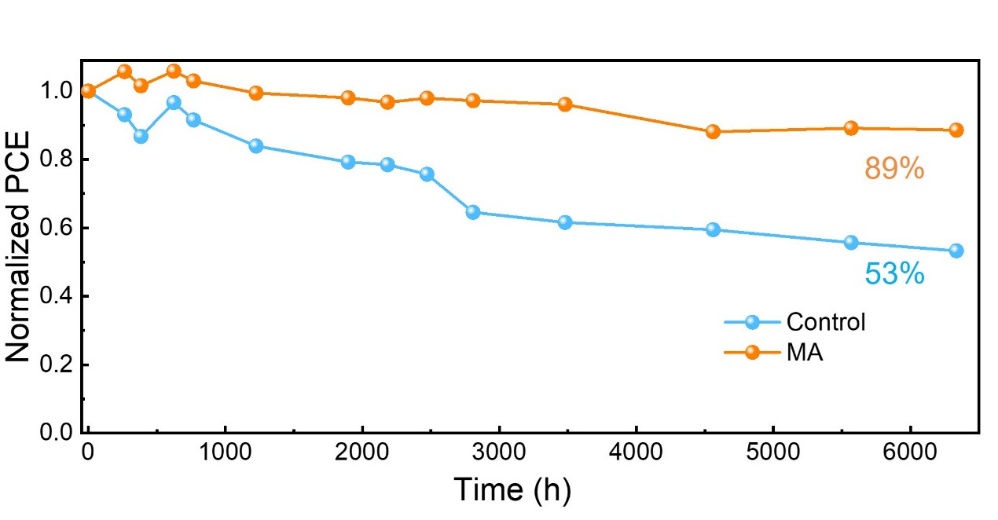
**

**Fig. S21** The stability of FPSCs under the N_2_ atmosphere


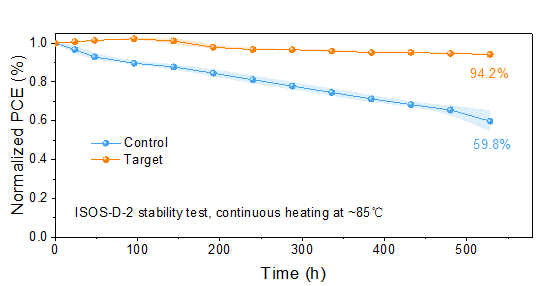


**Fig. S22** Normalized PCE of unencapsulated devices aged following the ISOS-D-2 standard

**Fig. S23** Normalized PCE for FPSCs after bending at different curvature radii for 1000 cycles

**Fig. S24** **a** Top-view and **b** Cross-sectional SEM images of control and target perovskite films after bending

**Table S1** The fitting parameters for time-resolved PL of the control and PTHM-modified perovskite films on SAM

| Sample | τ_1_ [ns] | A_1_ | τ_2_ [ns] | A_2_ | τ_avg_ [ns] |
| --- | --- | --- | --- | --- | --- |
| Control | 3.64 | 15431.71 | 76.73 | 0.61 | 3.69 |
| With MA | 6.90 | 105.18 | 294.71 | 0.47 | 52.93 |

$$\tau_{ave}=\frac{\sum A_{i}\tau_{i}^{2}}{\sum A_{i}\tau_{i}}$$

Fit the time-resolved PL decay curves to a biexponential decay function:

$$f\left( t \right)=A_{1}exp\left( \frac{-t}{\tau_{1}} \right)+A_{2}exp\left( \frac{-t}{\tau_{2}} \right)+B$$

**Table S2** Performance parameters of reported fPSCs

| Device structure | Initial PCE (%) | Bending cycles | Bending radius  (mm) | % of initial PCE | Time (h) | % of initial PCE | Refs. |
| --- | --- | --- | --- | --- | --- | --- | --- |
| PEN/ITO/MeO-2PACz/perovskite/C_60_/BCP/Ag | 25.03 | 3000 | 10 | 90.0 | 1850 | 90.0 | This work |
| PEN/ITO/2PACz/perovskite/C_60_/BCP/Cu | 25.01 | 3000 | 8 | 90.0 | 1000 | 85.0 | [S1] |
| PI/ITO//NiOx-SAM/perovskite/C60/BCP/Ag | 25.45 | 20000 | 5 | 97.3 | 1000 | 90.6 | [S2] |
| PEN/ITO/2PACz/perovskite/C60/BCP/Ag | 25.11 | 5000 | 3 | 92.6 | 1000 | 94 | [S3] |
| PEN/ITO/NiOx/PTAA/perovskite/PCBM/BCP/Ag | 23.7 | 10000 | 5 | 86 | 800 | 95 | [S4] |
| PET/ITO/2PACz/perovskite/C_60_/BCP/Ag | 23.0 | 5000 | 4 | 90.2 | 2000 | 81.3 | [S5] |
| PEN/ITO/SAM/perovskite/C_60_/BCP/Ag | 25.1 | 5000 | 5 | 93.0 | 400 | 87.0 | [S6] |
| PEN/ITO/PTAA/perovskite/C_60_/BCP/Cu | 23.0 | 10000 | 4 | 87.6 | 1000 | 85.6 | [S7] |
| PEN/ITO/SnO_2_/FAPbI_3_/PEAI/HTM/Ag | 24.19 | 4000 | 6 | 95.0 | 100 | 95 | [S8] |
| PEN/ITO/SnO_2_/Perovskite/Spiro-OMeTAD/Au | 24.43 | 10000 | 3 | 94.1 | 1000 | 90.0 | [S9] |
| PEN/ITO/SnO_2_/perovskite/PEAI/spiro-OMeTAD/Ag | 22.1 | 10000 | 2 | 83.0 | 200 | 87.0 | [S10] |
| PEN/ITO/SnO_2_/perovskite/spiro-OMeTAD/Au | 24.2 | 6000 | 5 | 95.6 | 200 | 90 | [S11] |

**Supplementary References**

1. Y. Wang, W. Chang, W. You, H. Xue, Y. Zhou et al., Constructing synergistic interactions between multi-hydroxyl molecules and perovskite to alleviate mechanical-thermal mismatch for achieving high-performance flexible solar cells. Angew. Chem. Int. Ed. **64**(40), e202512376 (2025). <https://doi.org/10.1002/anie.202512376>
2. W. Cai, J. Wang, X. Zhu, B. Jiao, L. Yang et al., *In situ* dual-region selective anchoring of zwitterionic gel enables efficient and mechanically durable flexible perovskite solar cell. Adv. Energy Mater. e05869 (2025). <https://doi.org/10.1002/aenm.202505869>
3. X. Sun, J. Gong, Q. Liu, D. Gao, X. Yu et al., Molecular tailoring of self-assembled monolayers *via* polar ether linker for highly efficient and mechanically robust flexible perovskite solar cells. Adv. Mater. e19365 (2025). <https://doi.org/10.1002/adma.202519365>
4. J. Jin, Z. Zhu, Y. Ming, Y. Zhou, J. Shang et al., Spontaneous bifacial capping of perovskite film for efficient and mechanically stable flexible solar cell. Nat. Commun. **16**(1), 90 (2025). <https://doi.org/10.1038/s41467-024-55652-6>
5. S. Zhu, X. Jin, W. Tan, Y. Zhang, G. Zhao et al., Multiple dynamic hydrogen bonding networks boost the mechanical stability of flexible perovskite solar cells. Adv. Funct. Mater. **34**(48), 2408487 (2024). <https://doi.org/10.1002/adfm.202408487>
6. X. Tong, L. Xie, J. Li, Z. Pu, S. Du et al., Large orientation angle buried substrate enables efficient flexible perovskite solar cells and modules. Adv. Mater. **36**(38), e2407032 (2024). <https://doi.org/10.1002/adma.202407032>
7. H. Zhang, Y. Bi, Y. Wang, C. Liu, N. Chen et al., Manipulating crystallization kinetics of perovskites by acetylsalicylic acid for efficient and stable two-step inverted flexible perovskite solar cells. Chem. Eng. J. **518**, 164708 (2025). <https://doi.org/10.1016/j.cej.2025.164708>
8. Z. Yang, J. Wei, Y. Liu, Y. Jiang, L. Liu et al., Radical p-doping spiro-OMeTAD for efficient, stable and self-healing flexible perovskite solar cells. Adv. Mater. **37**(27), 2417404 (2025). <https://doi.org/10.1002/adma.202417404>
9. Z. Li, C. Jia, Z. Wan, J. Cao, J. Shi et al., Boosting mechanical durability under high humidity by bioinspired multisite polymer for high-efficiency flexible perovskite solar cells. Nat. Commun. **16**(1), 1771 (2025). <https://doi.org/10.1038/s41467-025-57102-3>
10. Q. Sun, X. Meng, G. Liu, S. Duan, D. Hu et al., SnO_2_ surface modification and perovskite buried interface passivation by 2, 5-furandicarboxylic acid for flexible perovskite solar cells. Adv. Funct. Mater. **34**(45), 2404686 (2024). <https://doi.org/10.1002/adfm.202404686>
11. X. Zhu, Y. Li, Q.-Z. Li, N. Wang, S. Yang et al., Restrictive heterointerfacial delamination in flexible perovskite photovoltaics using a bifacial linker. Adv. Mater. **37**(13), e2419329 (2025). <https://doi.org/10.1002/adma.202419329>
